# Prepared for Mission? A Survey of Medical Personnel Training Needs Within the International Committee of the Red Cross

**DOI:** 10.1007/s00268-018-4651-5

**Published:** 2018-05-02

**Authors:** Frederike J. C. Haverkamp, Harald Veen, Rigo Hoencamp, Måns Muhrbeck, Johan von Schreeb, Andreas Wladis, Edward C. T. H. Tan

**Affiliations:** 1 Department of Surgery (internal postal code 618), Radboudumc, P.O. Box 9101, 6500 HB Nijmegen, The Netherlands; 2 World Health Organization, Geneva, Switzerland; 3 Department of Surgery, Alrijne Medical Centre Leiderdorp, Leiderdorp, The Netherlands; 4 Department of Surgery, Leiden University Medical Centre, Leiden, The Netherlands; 5 Ministry of Defence, Utrecht, The Netherlands; 6 Department of Surgery, Linköping University, Gamla Övägen 25, 603 79 Norrköping, Sweden; 7 Department of Clinical and Experimental Medicine, Linköping University, Gamla Övägen 25, 603 79 Norrköping, Sweden; 8 Department of Public Health Sciences, Karolinska Institutet, 171 77 Stockholm, Sweden; 9 International Committee of the Red Cross, 19 Avenue de la paix, 1202 Geneva, Switzerland; 10 Center for Disaster Medicine and Traumatology, Linköping University, Linköping, Sweden; 11 Department of Clinical and Experimental Medicine, Linköping University, Linköping, Sweden

## Abstract

**Background:**

Humanitarian organizations such as the International Committee of the Red Cross (ICRC) provide worldwide protection and medical assistance for victims of disaster and conflict. It is important to gain insight into the training needs of the medical professionals who are deployed to these resource scarce areas to optimally prepare them. This is the first study of its kind to assess the self-perceived preparedness, deployment experiences, and learning needs concerning medical readiness for deployment of ICRC medical personnel.

**Methods:**

All enlisted ICRC medical employees were invited to participate in a digital questionnaire conducted during March 2017. The survey contained questions about respondents’ personal background, pre-deployment training, deployment experiences, self-perceived preparedness, and the personal impact of deployment.

**Results:**

The response rate (consisting of nurses, surgeons, and anesthesiologists) was 54% (153/284). Respondents rated their self-perceived preparedness for adult trauma with a median score of 4.0 on a scale of 1 (very unprepared) to 5 (more than sufficient); and for pediatric trauma with a median score of 3.0. Higher rates of self-perceived preparedness were found in respondents who had previously been deployed with other organizations, or who had attended at least one master class, e.g., the ICRC War Surgery Seminar (*p* < 0.05). Additional training was requested most frequently for pediatrics (65/150), fracture surgery (46/150), and burns treatment (45/150).

**Conclusion:**

ICRC medical personnel felt sufficiently prepared for deployment. Key points for future ICRC pre-deployment training are to focus on pediatrics, fracture surgery, and burns treatment, and to ensure greater participation in master classes.

**Electronic supplementary material:**

The online version of this article (10.1007/s00268-018-4651-5) contains supplementary material, which is available to authorized users.

## Introduction

Our world is continuously being afflicted by disasters and political or armed conflict. Humanitarian aid organizations strive for improvement of the welfare and happiness of people or the alleviation of suffering and are essential for the protection and survival of victims of such disasters and wars. The International Red Cross and Red Crescent Movement is the largest of the world’s humanitarian organizations. The International Committee of the Red Cross (ICRC), based in Geneva, Switzerland, provides medical assistance to victims of war and other situations of violence, by deploying medical staff of various disciplines to conflict zones around the world.

An ICRC surgical team consists of an anesthesiologist, a surgeon, a surgical nurse, a ward nurse, and a physiotherapist. It is important this team is well prepared for giving (medical) care in austere environments [1–3]. All ICRC medical personnel are obliged to have (1) a valid medical or nursing degree and license to practice, (2) at least 2–5 years of experience in their field of expertise, and (3) a valid certificate from either Advanced Trauma Life Support (ATLS) or the European Trauma Course (ETC), in the case of anesthesiologists and surgeons. The ICRC continually organizes courses for their employees and has also recently defined best practices for clinical management of limb injuries in disasters and conflicts [4].

The ICRC is aiming to improve the setup of pre-deployment training for its teams. Gaining insight into the training and educational needs of their medical personnel prior to deployment is essential in delivering appropriate training and education. Current literature emphasizes the importance of standardized pre-deployment training programs for comparable organizations [2, 5–10]. Furthermore, the literature concerning military deployments reveals that pediatric patients make up a considerable proportion of the workload in conflict zones, representing 5.2–18% of all patients, and mostly requiring multiple surgical procedures per patient; correspondingly, more extensive pre-deployment training concerning the management of such patients has been requested by military medical teams [11–17]. The Emergency Medical Teams Initiative at the World Health Organization has defined standards for deployed teams intending to operate in disaster zones, which are currently being rolled out globally [18]. Based on this initiative, a 3-step training model has been proposed to ensure that deployed personnel are sufficiently prepared. Step 2 of this model is the adaptation of clinical skills to various contexts such as austere settings [19].

The aim of this study is to assess the self-perceived preparedness, deployment experiences, and learning needs concerning medical preparation for deployment of ICRC medical personnel, with a focus on the treatment of pediatric patients. With this information, recommendations can be made to the ICRC on how to optimize pre-deployment training to ensure that they meet both individual and institutional needs.

## Materials and methods

### Study design and selection of participants

This study was approved by ICRC headquarters in Geneva, Switzerland. Approval by an ethical committee was not required according to Dutch legislation [20]. A cross-sectional quantitative study was conducted in the form of a digital questionnaire. All enlisted ICRC medical employees as of February 2017 (284) were invited to participate by e-mail. The survey was sent out in March 2017, and participants were given 6 weeks to respond. Reminders were sent after 2 and 4 weeks. Participation in the survey was voluntary. Respondents consented to participation by submitting their response. The completed responses were rendered anonymous.

### Content of the questionnaire

The survey design was based on similar surveys by the armed forces and other sources in the current literature [5–7, 10, 21, 22] and was reviewed by specialists in military and emergency surgery and adapted accordingly (see Online Resource 1: the questionnaire). The survey contained questions about the personal background of the medical personnel, pre-deployment training, deployment experiences, self-perceived preparedness, and aftercare. Most questions required answering with either a 5-point Likert scale or a nominal scale, but several open questions were also included [23].

### Analysis

IBM SPSS Statistics (version 22, IBM Corporation, Armonk, New York) was used for all descriptive and comparative statistical analyses. Comparisons between groups are made by means of Chi-square tests, Mann–Whitney *U* tests, and Kruskal–Wallis analyses, and an *α* level of 0.05 was used.

## Results

### General information

The response rate for this survey was 54% (153/284). Not all questions were answered by each respondent. Demographic and background characteristics of the respondents are displayed in Table 1. The distribution in terms of profession did not significantly differ between respondents and non-respondents. Besides their deployments with the ICRC, 62.7% (96/153) of the respondents had previously also been deployed with other organizations such as Médecins Sans Frontières (MSF), while 20.9% (32/153) with the armed forces. During the previous 2 years, 45.8% (70/153) of the respondents had sporadically been involved in pediatric surgery; and 23.5% (36/153) had not been involved in any pediatric surgical procedures over the previous 2 years.

**Table 1:** Demographic and background characteristics

Characteristics	Frequency reported	Percentage *N* = 153
Gender		
Male	93	60.8%
Female	60	39.2%
Mean age (years)	47.6	SD 10.5 min 29–max 70
Profession		
Anesthetist	36	23.5%
General practitioner	1	0.7%
Emergency room physician	7	4.6%
Infectiologist	1	0.7%
Orthopedic surgeon	3	2.0%
Nurse	59	38.6%
Senior medical officer	1	0.7%
Surgeon	42	27.5%
Other	3	2.0%
Median period of board registration as medical specialist (years)	15	IQR 15.3
Median period of master’s degree (years)	24	IQR 18.0
Median experience in sub specialization (years)	13	IQR 18.5
Median number of deployments with the ICRC	3	IQR 4.5
Median duration of the ICRC deployments (weeks)	12	IQR 16.0

### Preparation for deployment with the ICRC

The number of respondents who participated in basic medical courses and master classes as preparation for deployment is listed in Tables 2 and 3. Twenty-seven (18%; 27/150) respondents did not attend either a basic course, or a master class. Respondents who attended at least one master class before deployment scored their self-perceived preparedness for adult and pediatric trauma higher than respondents who had not attended any (*p* < 0.05; for adults, mean rank 80.4 vs. 65.7; for pediatrics, mean rank 79.9 vs. 63.8). The same higher rating following master class attendance also applied to their medical pre-deployment training, knowledge, and skills (*p* < 0.05; mean rank 80.9 vs. 61.9).

**Table 2:** Participation in basic medical courses

Basic courses^(suitable for)^	Number of respondents that participated^h^	Percentage
Advanced Trauma Life Support (ATLS)^(a, b, c, d, e, f, g) i^	56/90	62.2%
Advanced Life Support (ALS)^(a, b, c, d, e, f, g)^	52/149	34.9%
Advanced Pediatric Life Support (APLS)^(a, b, c, d, e, f, g) j^	19/90	21.1%
European Pediatric Advanced Life Support (EPALS)^(a, b, c, d, e, f, g)^	5/149	3.4%
Other^k^	42/150	28.0%
None	48/150	32.0%

^a^Anaesthesiologist; ^b^general practitioner; ^c^emergency room physician; ^d^infectiologist; ^e^orthopaedic surgeon; ^f^registered nurse; ^g^surgeon

^h^Respondents were allowed to mark multiple courses

^i^13 nurses participated as participant or observer

^j^4 nurses participated as participant or observer

^k^Courses that were mentioned more than once were Advanced Cardiovascular Life Support (*n* = 4) and Basic Life Support (*n* = 5)

**Table 3:** Participation in master classes

Master class^(suitable for)^	Number of respondents that participated^j^	Percentage	Median rating for general preparation (IQR)^k^
Definitive Surgical Trauma Care (DSTC) Course^(f, g, i)^	10/104	9.6%	4.0 (1.0)
Definitive Anesthetic Trauma Care (DATC) Course^(a, g)^	2/95	2.1%	3.5 (0.0)
Definitive Surgical Trauma Skills (DSTS)^(f, i)^	2/45	4.4%	4.0 (0.0)
Surgical Training for Austere Environments (STAE)^(f, i)^	7/45	15.6%	4.0 (1.0)
Military Operational Surgical Training (MOST)^(a, c, f, g, i)^	1/147	0.7%	2.0 (0.0)
Emergency War Surgery Course (EWSC)^(a, f, g, i) l^	6/45	13.3%	4.0 (0.75)
Advanced Trauma Operative Management (ATOM)^(c, f, i)^	1/45	2.2%	5.0 (0.0)
Advanced Surgical Skills for Exposure in Trauma (ASSET)^(f, i)^	0/45	0%	Not applicable
Health Emergencies in Large Populations (HELP) course^(a, b, c, d, e, f, g, h, i)^	8/150	5.3%	4.0 (1.0)
ICRC War Surgery Seminar “The management of patients with war wounds”^(a, c, f, g, i)^	69/147	47.0%	4.0 (2.0)
European Trauma Course (ETC)^(a, b, c, d, e, f, g, h, i)^	7/150	4.7%	4.0 (1.0)
Other	20/150	13.3%	Not applicable
None	60/150	40.0%	Not applicable

*IQR* interquartile range

^a^Anaesthesiologist; ^b^general practitioner; ^c^emergency room physician; ^d^facilitator; ^e^infectiologist; ^f^orthopaedic surgeon; ^g^registered nurse; ^h^senior medical officer; ^i^surgeon

^j^Respondents were allowed to mark multiple courses

^k^Scale: 1 not important at all–5 absolutely essential

^l^1 nurse participated as participant or observer

There were various topics on which respondents stated they would like to have additional training (Table 4); 12% (18/150) of respondents did not feel the need for additional training in any aspect. The most frequently requested topic was pediatrics (*N* = 65, 43.3%), and subanalysis between professions showed that nurses requested additional training in this topic more frequently (*N* = 35, 53.8%) than surgeons (*N* = 10, 15.4%) and anesthesiologists (*N* = 12, 18.5%, *p* < 0.05). Anesthesiologists requested additional training on fewer topics than surgeons and nurses (*p* < 0.05). Furthermore, respondents who had been previously deployed with the armed forces or another organization requested additional training for fewer topics than respondents who had only been deployed with the ICRC (*p* < 0.05).

**Table 4:** Topics requested for additional training

Topics	Frequency reported^a^	Percentage (*N* = 150)
Fracture surgery	46	30.7%
Soft tissue surgery	21	14.0%
Burn treatment	45	30.0%
Gastro intestinal surgery	12	8.0%
Pediatrics	65	43.3%
Thorax surgery	26	17.3%
Vascular surgery	29	19.3%
Plastic (reconstructive) surgery	30	20.0%
Urology	11	7.3%
Neurosurgery	26	17.3%
Obstetrics/gynecology	28	18.7%
Ophthalmic surgery	10	6.7%
Maxillofacial surgery	38	25.3%
Other	24	16%
No need for additional training	18	12.0%

^a^Respondents were allowed to mark multiple topics

In addition, the group of respondents that had previously been deployed with organizations other than the ICRC rated their self-perceived preparedness for pediatric trauma, and their medical training, knowledge, and skills higher (*p* < 0.05). The ratings for self-perceived preparedness are displayed in Table 5. Concerning adult trauma, 92.7% (139/150) of the respondents rated their self-perceived preparedness for deployment as sufficient (rating 3–5 on a scale of 1 being very unprepared–5 being more than sufficiently prepared); self-perceived preparedness concerning pediatric trauma was rated as sufficient by 62% (93/150).

**Table 5:** Self-perceived preparedness

Feeling of preparedness	Median score^a^	IQR
Self-perceived preparedness rated prior to deployment		
For adult trauma	4.0	1.0
For pediatric trauma	3.0	2.0
Self-perceived preparedness rated after/during deployment		
For adult trauma	4.0	1.0
For pediatric trauma	3.0	2.0
Rating of own medical training, knowledge and skills regarding injuries treated	4.0	1.0
Rating of medical training, knowledge and skills of colleagues		
In general	4.0	1.0
Regarding pediatrics	3.0	2.0

*IQR* interquartile range

^a^Scale: 1 very unprepared–5 more than sufficient

Respondents who had more experience with pediatric surgery prior to deployment rated their self-perceived preparedness for pediatric surgery higher (*p* < 0.05). Nurses scored their self-perceived preparedness for pediatric surgery and their medical training, knowledge, and skills lower than surgeons (*p* < 0.05).

Cooperation with the armed forces in pre-deployment training was supported by 41.4% (60/145) of the respondents.

### Deployment experiences

Exposure to adult trauma during the last ICRC deployment ranged from once a week to every day for 92% (138/150) of the respondents. Concerning pediatric trauma, 42.7% (64/150) reported having been exposed to this frequently.

Table 6 lists the median satisfaction with equipment available at various treatment locations during deployment. Respondents who had previously been deployed more often with the ICRC were better satisfied with the equipment available for adult treatment in the operating room and during follow-up (*p* < 0.05).

**Table 6:** Satisfaction with equipment on different locations

Treatment location	Median score^a^	IQR
Prehospital		
For adult patients	2.0	3.0
For pediatric patients	1.0	3.0
In the emergency room		
For adult patients	3.0	2.0
For pediatric patients	2.0	2.0
In the operation room		
For adult patients	4.0	2.0
For pediatric patients	3.0	3.0
In the intensive care unit		
For adult patients	2.0	3.0
For pediatric patients	2.0	3.0
During follow-up		
For adult patients	3.0	2.0
For pediatric patients	3.0	2.0

*IQR* interquartile range

^a^Scale: 1 very dissatisfied–5 very satisfied

Regarding systematic methods being used to facilitate the transfer of information about a patient between medical services, 63.8% (95/149) of the respondents reported that during deployment no systematic methods were used when transferring information. In cases where a systematic method was used, MIST (Mechanism of injury, Identified injury, Signs, Therapy) was used most frequently (reported to be used by 18.8%; 28/149). Furthermore, 43.3% (65/150) reported that they never received adequate information in time from prehospital medical services to prepare the crash room; and 32% (48/150) reported receiving adequate information in less than 50% of cases of an incoming trauma patient.

Telemedicine is described as the delivery of healthcare services by all healthcare professionals using information and communication technologies. Most respondents (62%; 93/150) reported that there was no type of telemedicine available during their ICRC deployment, but when available, the most frequently used medium was phone (reported by 15.3%; 23/150).

The availability of a referral center for pediatric surgery during deployment was reported by 18.7% (28/150). A ground ambulance was most frequently (47.4%; 18/38) reported as the means of transport for reaching the referral center, followed by own transportation (18.4%; 7/38), and taxi (15.8%; 6/38). With the quickest means of transport, 42.9% (12/28) of respondents reported that a patient was able to reach the referral center within 1 h; 10.7% (3/28) reported that it would take more than 1 h; and 28.6% (8/28) reported that it would take more than 2 h.

### Personal impact

The need for an independent coach, to talk about their experiences during deployment, was reported by 44.7% (67/150) of the respondents. Of these, 32.8% (22/67) actually talked to an independent coach. The survey identified that 78% (117/150) of the respondents felt the need for debriefing with direct colleagues on their experiences during deployment; while 76.9% (90/117) of those actually engaged in this debriefing. Concerning professional help, 41.3% (62/150) felt the need for this during deployment, and 67.7% (42/62) actually received such help. The need for aftercare was not significantly lower among respondents who rated their own professional knowledge and skills higher, or who had undertaken previous deployments for organizations other than the ICRC.

Most respondents (78%; 117/150) reported a positive effect of their deployment on their professional knowledge and skills; and 84% (126/150) reported a positive effect on their personal development. Nearly, one-quarter of respondents (23.3%; 35/150) reported a negative effect of their deployment on their home life.

### Limitations and strengths

This study is the first of its kind to identify the learning needs and self-perceived preparedness of ICRC medical professionals. Although this questionnaire was not validated, it was based on existent literature and was thoroughly reviewed and criticized by multiple experts in the field of military and emergency surgery [5–7, 10, 21, 22].

All inherent survey limitations could also be said to have played a role in this survey. A response rate of 54% was achieved, which resulted in a margin of error of 5% with a confidence interval of 95. Response bias was limited by the anonymity of the survey, expecting this would decrease the pressure for respondents to give answers that are assumed to be socially or politically desirable.

This survey focused on different professions as one group of respondents, but, in reality, their needs in terms of pre-deployment training may differ. This should be taken into account during further research in the form of an updated sequential survey on this topic.

## Discussion

Our study results will provide the ICRC with information to guide their future pre-deployment training and focus it on the learning needs of their medical personnel. It is noteworthy that over 90% of the respondents rated their self-perceived preparedness for adult trauma as sufficient. Even though the respondents represent an experienced group of medical professionals, they still noted the need for additional training in various aspects, and a significant need for aftercare. The most frequently requested topics for additional training were pediatrics, fracture surgery, and burns treatment. Most respondents had attended at least one basic course or master class as preparation for their deployment with the ICRC. This seemed to significantly improve self-perceived preparedness.

A survey among Dutch deployed (military) surgeons and anesthesiologists revealed that they rated the DSTC or DATC courses as very useful [7]. Waxman et al. [24] state that during humanitarian aid operations, wound management should follow ICRC guidelines, which is also a focus of the ICRC War Surgery Seminar. A greater focus on attending these courses in the pre-deployment training could be considered.

Most respondents had little experience of pediatric surgery in the non-deployed setting, but during deployment, exposure to this patient population was significant and in most cases, there was no referral center available for these patients. The need for proper pre-deployment training regarding this patient population has previously been addressed in literature [13, 15, 16]. Strikingly, US army surgeons have reported a low need for additional training on pediatric trauma [5, 25]; and Dutch deployed (military) surgeons have rated their self-perceived medical expertise relatively high on the topics of fracture surgery, burns treatment, and pediatrics [7]. It should be noted that Dutch trauma surgeons perform many orthopedic procedures in their daily practice, which could explain the differences in the need for additional training concerning fracture surgery. Further epidemiological studies are required to identify the medical needs of pediatric patients during ICRC deployments and to specify which skills are essential to adequately manage these patients.

The results of this survey revealed that nurses felt less prepared for deployment than surgeons and anesthesiologists, which is in line with the findings of another questionnaire among military nurses [2]. Pre-deployment training should, it is suggested, be focused on all professions involved in the deployed medical team and should clarify their specific roles.

A significant group of respondents supported cooperation with the armed forces in pre-deployment training. It has been acknowledged that military systems could provide substantial assistance to civil societies at times of crises [26, 27]. This cooperation remains controversial, specifically in regard to the independence, impartiality, and neutrality of humanitarian aid organizations [28].

In most cases of an incoming trauma, no systematic methods were used for the transfer of information about the patient. Interviews among Swedish military doctors and nurses revealed that a structured means of communication was seen as part of functioning emergency care [29]. Yet there remains no international consensus on this matter, and evidence for the usefulness of handover mnemonics is inconclusive [30].

It is not uncommon to be the sole medical professional of a certain discipline during deployment. Telemedicine could therefore be an important means of taking a multidisciplinary approach [31–34].

A need for some type of aftercare was reported by most respondents, but was not always fulfilled. Specific pre-deployment briefings about stress management, the availability of a consistent contact person during deployment, the discussing of deployment experiences with colleagues, post-deployment follow-up, and prior military courses or experiences have all been highly appreciated as contributors to mental readiness for deployment by other deployed medical professionals [5, 7, 21, 22]. However, our data did not show a statistical decrease in the need for aftercare among respondents who had had prior deployment preparation experiences. The importance of debriefing and discussing experiences with colleagues before, during, and after deployment should not be underestimated.

It is recommended for the ICRC and similar organizations which provide medical care in conflict zones, to adequately prepare their deployed medical personnel by strongly encouraging participation in master classes such as the ICRC War Surgery seminar, and in specific courses focusing on pediatric trauma. Nurses who responded to this questionnaire reported a lower feeling of self-perceived preparedness for deployment than surgeons and anesthesiologists; the ICRC has now implemented a 2-month onboarding mission for nurses, which already had been realized for surgeons and anesthesiologists. During this onboarding mission, the new medical employee is supplementary, and therefore gets the chance to become familiar with the working conditions during deployment. Furthermore, the accessibility of telemedicine during deployment, and the indications to use it, should be highlighted more prior to deployment. Likewise, the importance and availability of debriefing procedures during and after deployment should be emphasized.

## Conclusion

Conclusively, medical personnel of the ICRC who responded to our questionnaire stated that they felt sufficiently prepared for deployment. Attendance on pre-deployment master class was a condition of a greater sense of preparedness. More than 75% of the respondents reported a need for debriefing with direct colleagues, emphasizing the high impact of humanitarian deployments. Based on the ICRC medical employees’ learning needs, recommendations (focus on pediatrics, fracture surgery and burns treatment) are made for the content of future ICRC pre-deployment training. This could further improve the medical preparedness of ICRC personnel and could, by extension, benefit the victims of war treated by its staff. Further research in this field could focus on repetitive analysis of ICRC medical employees over time, taking into consideration their different professions.

## Electronic supplementary material

Below is the link to the electronic supplementary material.

Supplementary material 1 (PDF 382 kb)
