# Supplementary material for: Prepared for Mission? A Survey of Medical Personnel Training Needs Within the International Committee of the Red Cross
Source: World J Surg. 2018 May 2;42(11):3493–500. doi: 10.1007/s00268-018-4651-5 (PMC6182760; doi:10.1007/s00268-018-4651-5)
Supplement: Supplementary file 1 — Supplementary material 1 (PDF 382 kb) [file 268_2018_4651_MOESM1_ESM.pdf]

Online Resource 1: the questionnaire

Title: Prepared for mission? A survey of medical personnel training needs within the International Committee of the Red Cross

Journal: World Journal of Surgery

Authors:
Frederike J.C. Haverkamp, BSc[1], corresponding author,
frederike.haverkamp@radboudumc.nl
Harald Veen, MD
Rigo Hoencamp, MD, PhD
Måns Muhrbeck, MD
Johan von Schreeb, MD, PhD
Andreas Wladis, MD, PhD
Edward C.T.H. Tan, MD, PhD

Affiliations:
[1] Department of Surgery, Radboudumc, Nijmegen, the Netherlands
Postal address: Afdeling Heelkunde 618, Frederike Haverkamp, Edward Tan, Postbus 9101, 6500 HB Nijmegen

ICRC INTERNATIONAL COMMITTEE OF THE RED CROSS

This survey contains questions about self-efficacy and pre-deployment training, with a focus on paediatric surgery preparation. Your participation in this survey is voluntary. Your answers will be made anonymous and the outcome of this survey will be used only for research and study intentions.

It takes about 10 to 15 minutes to complete this survey. Your unfinished response can be saved and resumed later. There is an explanation field added to most of the questions. You can use it in case you feel the need to elaborate your answer, but this is not mandatory.

LINKÖPING UNIVERSITY Radboudumc university medical center Karolinska Institutet

1.1 General information

1) Gender
   a) Male
   b) Female

2) Age (years):

3) Profession/Position name:
   a) Anaesthetist
   b) General practitioner
   c) Emergency Room physician
   d) Facilitator
   e) Infectiologist
   f) Orthopaedic Surgeon
   g) Registered nurse
      i) Department/position:
         (1) Emergency Department nurse
         (2) Intensive Care Unit nurse
         (3) Surgical nurse
         (4) Teaching nurse
         (5) Ward nurse
         (6) Other (specify)
   h) Senior medical officer
   i) Surgeon
   j) Other (specify)

4) Sub specialization (if applicable):

5) Years of experience in sub specialization (if applicable):

6) Date (year) of M.D. degree (if applicable):

7) Date (year) of board registration as a medical specialist (if applicable):

8) Experience with paediatric surgery during the last 2 years:
   a) Not involved in any paediatric surgical procedures
   b) Sporadically involved in paediatric surgical procedures
   c) Involved in paediatric surgical procedures on a monthly basis
   d) Involved in paediatric surgical procedures on a weekly basis
   e) Involved in paediatric surgical procedures on a daily basis

1.2 ICRC deployment(s)

Please first fill in the specifications of your latest deployment

1) How many deployments have you done with the ICRC?

[Respondents could fill in specifications of each deployment]

1) Starting date (year):
   a) I do not remember

2) Duration:
   a) Ongoing
   b) I do not remember

3) Location:
   a) I do not remember

4) In which setting did you work during this deployment?
   a) Type I – ICRC independent hospital
   b) Type II – ICRC substitution program
   c) Type III – ICRC support program
   d) Type IV – ICRC monitored program
   e) I do not remember
   f) Other:

5) Brief job description:

6) Were you involved in paediatric surgery during this deployment?
   a) Yes
   b) No

1.3 Deployment(s) with the armed forces

Please first fill in the specifications of your latest deployment

1) How many deployments have you done with the armed forces?

[Respondents could fill in specifications of each deployment]

1) Starting date (year):
   a) I do not remember

2) Duration:
   a) Ongoing
   b) I do not remember

3) Location:
   a) I do not remember

4) In which setting did you work during this deployment?
   a) Role 1 medical treatment facility
   b) Forward surgical team
   c) Role 2 medical treatment facility
   d) Role 3 medical treatment facility
   e) Role 4 medical treatment facility
   f) Other (specify):
   g) I do not remember
   h) Other:

5) Brief job description:

6) Were you involved in paediatric surgery during this deployment?
   a) Yes
   b) No

1.4 Other deployment(s)

Please first fill in the specifications of your latest deployment

1) How many deployments have you done with other organisations?

[Respondents could fill in specifications of each deployment]

1) Organisation:
   a) Médecins Sans Frontières (MSF)
   b) Other:

2) Starting date (year):
   a) I do not remember

3) Duration:
   a) Ongoing
   b) I do not remember

4) Location:
   a) I do not remember

5) In which setting did you work during this deployment?
   a) Battle field
   b) Field hospital
   c) Hospital boat
   d) Other (specify):
   e) I do not remember
   f) Other:

6) Brief job description:

7) Were you involved in paediatric surgery during this deployment?
   a) Yes
   b) No

1.5 Pre-deployment preparations

The following questions only refer to your last (or current) deployment with the ICRC

1) Which basic courses have you done prior to your last ICRC deployment?
    a) Advanced Trauma Life Support (ATLS)
    b) Advanced Life Support (ALS)
    c) Advanced Paediatric Life Support (APLS)
    d) European Paediatric Advanced Life Support (EPALS)
    e) ICRC Emergency Room Trauma Course (ERTC)
    f) None
    g) Other:

2) What additional courses or trainings have you done prior to your last ICRC deployment and what year (if you remember) did you take them?
    a) Course name:
        i) Definitive Surgical Trauma Care (DSTC) Course
            (1) Date (year)
        ii) Definitive Anaesthetic Trauma Care (DATC) Course
            (1) Date (year)
        iii) Definitive Surgical Trauma Skills (DSTS)
            (1) Date (year)
        iv) Surgical Training for Austere Environments (STAE)
            (1) Date (year)
        v) Military Operational Surgical Training (MOST)
            (1) Date (year)
        vi) Emergency War Surgery Course (EWSC)
            (1) Date (year)
        vii) Advanced Trauma Operative Management (ATOM)
            (1) Date (year)
        viii) Advanced Surgical Skills for Exposure in Trauma (ASSET)
            (1) Date (year)
        ix) Health Emergencies in Large Populations (HELP) course
            (1) Date (year)
        x) ICRC Surgical Seminar "The management of patients with war wounds"
            (1) Date (year)
        xi) European Trauma Course (ETC)
            (1) Date (year)
        xii) Other (specify):
            (1) Date (year)

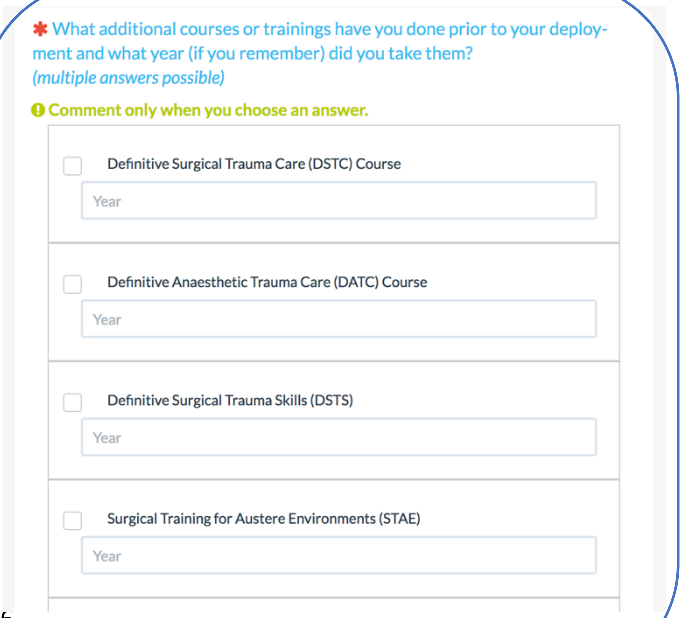

3) How would you value each course, with regard to your general preparation and your preparation for paediatric trauma?
   a) Rating for my general preparation:
      i) Not important at all
      ii) Of little importance
      iii) Of average importance
      iv) Very important
      v) Absolutely essential
      vi) No opinion

   b) Rating for my preparation for paediatric trauma:
      i) Not important at all
      ii) Of little importance
      iii) Of average importance
      iv) Very important
      v) Absolutely essential
      vi) No opinion

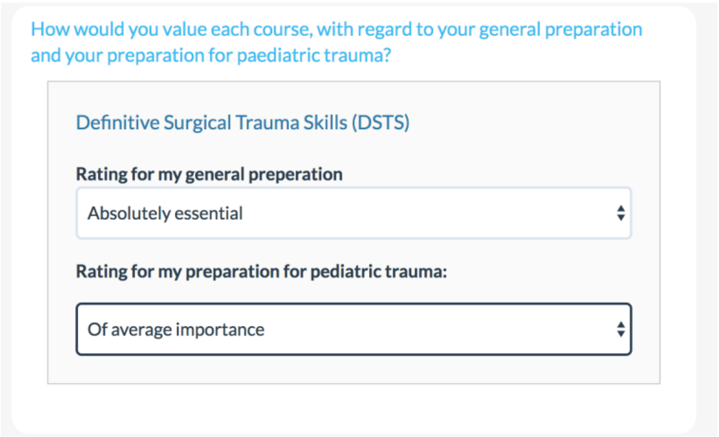

4) What did you value most during your preparation for your last ICRC deployment? Please explain why.

5) Please mark in which subject(s) you would have liked to get additional training prior to your last ICRC deployment (you are allowed to mark multiple subjects):
   a) Fracture surgery
   b) Soft tissue surgery
   c) Burn treatment
   d) Gastro intestinal surgery
   e) Paediatrics
   f) Thorax surgery
   g) Vascular surgery
   h) Plastic (reconstructive) surgery
   i) Urology
   j) Neurosurgery
   k) Obstetrics/Gynaecology
   l) Ophthalmic surgery
   m) Maxillofacial surgery
   n) Other:
   o) I did not feel the need for an additional training

6) Is there anything else you think is missing in the offered courses and trainings to prepare for deployments for the ICRC?

7) Are you pro or con a close cooperation with the armed forces in pre-deployment training?
   a) Pro, because:
   b) Con, because:
   c) No opinion

8) Prior to your last ICRC deployment, did you feel professionally prepared for paediatric trauma?
   a) Very unprepared
   b) Not enough
   c) Acceptable
   d) Sufficient
   e) More than sufficient
   f) I do not remember/No opinion
      Explanation:

9) Prior to your last ICRC deployment, did you feel professionally prepared for adult trauma?
   a) Very unprepared
   b) Not enough
   c) Acceptable
   d) Sufficient
   e) More than sufficient
   f) I do not remember/No opinion
      Explanation:

1.6 Deployment experiences

The following questions only refer to your last (or current) deployment with the ICRC.

1) How frequently were you exposed to paediatric trauma during your last ICRC deployment?
   a) Less than once a month
   b) Once a month
   c) A few times a month
   d) Once a week
   e) A few times a week
   f) Every day
   g) I do not remember
   Explanation:

2) How frequently were you exposed to adult trauma during your last ICRC deployment?
   a) Less than once a month
   b) Once a month
   c) A few times a month
   d) Once a week
   e) A few times a week
   f) Every day
   g) I do not remember
   Explanation:

3) Were your pre-deployment training, knowledge and skills sufficient regarding the injuries you have treated during your last ICRC deployment?
   a) Very insufficient
   b) Insufficient
   c) Fairly sufficient
   d) Sufficient
   e) More than sufficient
   f) I do not remember/No opinion
   Explanation:

4) In general, how do you consider the medical training, knowledge and skills of your colleagues during your last ICRC deployment?
   a) Very poor
   b) Below average
   c) Average
   d) Above average
   e) Excellent
   f) I do not remember/No opinion
   Explanation:

5) How do you consider the medical training, knowledge and skills of your colleagues during your last ICRC deployment regarding paediatric injuries?
   a) Very poor
   b) Below average
   c) Average
   d) Above average
   e) Excellent
   f) I do not remember/No opinion
   Explanation:

6) How do you rate your confidence in your skills regarding the treatment of paediatric patients compared to the treatment of adult patients during your last ICRC deployment?
   a) Much more confident in treating adult patients
   b) Slightly more confident in treating adult patients
   c) Equally confident in treating adult and paediatric patients
   d) Slightly more confident in treating paediatric patients
   e) Much more confident in treating paediatric patients
   f) I do not remember/No opinion
   Explanation:

7) In the following locations, were you satisfied about the equipment you had to your disposal to treat adult patients during your last ICRC deployment?
   a) Prehospital:
      i) Very dissatisfied
      ii) Dissatisfied
      iii) Neutral
      iv) Satisfied
      v) Very satisfied
      vi) Not applicable
   b) In the emergency room
   c) In the operation room
   d) In the intensive care unit
   e) During follow-up
   Explanation:

8) In the following locations, were you satisfied about the equipment you had to your disposal to treat paediatric patients during your last ICRC deployment?
   a) Prehospital:
      i) Very dissatisfied
      ii) Dissatisfied
      iii) Neutral
      iv) Satisfied
      v) Very satisfied
      vi) Not applicable
   b) In the emergency room
   c) In the operation room
   d) In the intensive care unit
   e) During follow-up

Explanation:

9) In how many of the incoming traumas did you receive adequate information in time from prehospital medical services to prepare the crash room?
   a) Never, because no prehospital medical care was provided
   b) Never (for any other reason)
   c) In less than 25% of the cases
   d) In 25-50% of the cases
   e) In 50-75% of the cases
   f) In more than 75% of the cases
   g) I do not remember
   Explanation:

10) Which systematic methods were used to transfer information about patients from other medical services to the emergency ward personnel? (Multiple answers possible)
   a) MIST (Mechanism of injury, Injuries sustained, Signs, Treatment and Trends in the vital signs)
   b) SBAR (Situation, Background, Assessment, Recommendation)
   c) RSVP (Reason, Story, Vital signs, Plan)
   d) No systematic methods were used
   e) Other (specify):
   f) I do not remember
   Explanation

11) How frequently was that method used when information about a patient was transferred?
   a) In less than 25% of the cases
   b) In 25%-50% of the cases
   c) In 50%-75% of cases
   d) In more than 75% of the cases
   e) I do not remember
   f) Explanation

12) If telemedicine was available during your last ICRC deployment: which types of telemedicine were available? (Multiple answers possible)
   a) Phone
   b) E-mail
   c) Fax
   d) Internet
   e) Other:
   f) No telemedicine was possible
   g) I do not remember
   Explanation:

13) Which type of telemedicine was used most frequently?
   a) Phone
   b) E-mail
   c) Fax
   d) Internet

e) Other:
f) I do not remember
   Explanation:

14) How frequently was telemedicine used in the treatment of a patient during your last ICRC deployment?
   a) In less than 25% of the cases
   b) In 25-50% of the cases
   c) In 50-75% of the cases
   d) In more than 75% of the cases
   e) I do not remember
   Explanation:

15) How frequently was the telemedicine of additional value for the treatment of a patient?
   a) In less than 25% of the cases when telemedicine was used for a patient
   b) In 25-50% of the cases when telemedicine was used for a patient
   c) In 50-75% of the cases when telemedicine was used for a patient
   d) In more than 75% of the cases when telemedicine was used for a patient
   e) I do not remember
   Explanation:

16) To what extend were the procedures you performed during your last (or current) ICRC deployment similar to the procedures you perform in a non-deployed setting?
   a) Not at all
   b) Slightly
   c) Somewhat
   d) Moderately
   e) Extremely
   f) I do not remember
   Explanation:

17) Was a referral centre for paediatric surgery available?
   a) Yes
   b) No
   c) I do not remember
   Explanation:

18) Which ways of transportation were available during your last ICRC deployment for the transfer of patients to a centre for paediatric surgery? (Multiple answers possible)
   a) Own transportation
   b) Ground ambulance
   c) Taxi
   d) Other (specify):
   e) I do not remember
   Explanation:

19) How fast could a patient arrive at a referral centre for paediatric surgery during your last ICRC deployment? (With the quickest mode of transportation)

a) Less than one hour
b) More than one hour
c) More than two hours
d) I do not remember
Explanation:

20) How frequently did you refer a patient to a centre for paediatric surgery during your last ICRC deployment?
a) Never
b) Incidentally
c) Monthly
d) Weekly
e) Daily
f) I do not remember
Explanation:

1.7 After deployment with the ICRC

The following questions only refer to your <u>last</u> deployment for the ICRC.

1) In question 16 “In advance of your deployment, did you feel professionally prepared for paediatric trauma?”, you answered you felt "… ". How would you rate your preparedness for paediatric trauma now after the deployment?
   a) Very unprepared
   b) Not enough
   c) Acceptable
   d) Sufficient
   e) More than sufficient
   f) I do not remember/No opinion
   Explanation:

2) In question 19 “In advance of your deployment, did you feel professionally prepared for adult trauma?”, you answered you felt "… ". How would you rate your preparedness for adult trauma now after the deployment?
   a) Very unprepared
   b) Not enough
   c) Acceptable
   d) Sufficient
   e) More than sufficient
   f) I do not remember/No opinion
   Explanation:

3) What impact did your last ICRC deployment have on your trauma management skills? Your trauma management skills:
   a) Much deteriorated
   b) Slightly deteriorated
   c) Did not change
   d) Slightly improved
   e) Much improved
   f) No opinion
   Explanation:

4) What impact did your last ICRC deployment have on your skills in your primary specialism? Your skills:
   a) Much deteriorated
   b) Slightly deteriorated
   c) Did not change
   d) Slightly improved
   e) Much improved
   f) No opinion
   Explanation:

1.8 Personal

The following questions only refer to your last deployment with the ICRC.

1) Did you feel the need for an independent “coach” (peer to peer), to talk about your experiences during your last ICRC deployment?
   a) Not at all
   b) Not really
   c) Undecided
   d) Somewhat
   e) Very much
   f) I do not remember/No opinion
   Explanation:

2) Did you, in fact, talk to an independent “coach” about your experiences during your last ICRC deployment?
   a) Yes
   b) No
   Explanation:

3) Did you ever feel the need to debrief with direct colleagues on your experiences during your last ICRC deployment?
   a) Not at all
   b) Not really
   c) Undecided
   d) Somewhat
   e) Very much
   f) I do not remember/No opinion
   Explanation:

4) Did you, in fact, debrief with direct colleagues on your experiences during your last ICRC deployment?
   a) Yes
   b) No
   Explanation:

5) Did you ever feel the need for professional help during your last ICRC deployment?
   a) Not at all
   b) Not really
   c) Undecided
   d) Somewhat
   e) Very much
   f) I do not remember/No opinion
   Explanation:

6) Did you, in fact, get professional help during your last ICRC deployment?
   a) Yes
   b) No
   Explanation:

7) What effect did your last ICRC deployment have on your professional knowledge and skills?
   a) Major negative effect
   b) Minor negative effect
   c) Neutral
   d) Minor positive effect
   e) Major positive effect
   f) No opinion
   Explanation:

8) What effect did your last ICRC deployment have on your personal development?
   a) Major negative effect
   b) Minor negative effect
   c) Neutral
   d) Minor positive effect
   e) Major positive effect
   f) No opinion
   Explanation:

9) What effect did your last ICRC deployment have on your situation at home?
   a) Major negative effect
   b) Minor negative effect
   c) Neutral
   d) Minor positive effect
   e) Major positive effect
   f) No opinion
   Explanation:

10) Regarding preparation (e.g. courses or training), what would you especially recommend for colleagues preparing for a mission in a conflict zone?

Thank you for your cooperation

If you want to stay informed about the results of this survey, please fill in your e-mail address:
